# Orphan Nuclear Receptor RORα Regulates Enzymatic Metabolism of Cerebral 24S-Hydroxycholesterol through CYP39A1 Intronic Response Element Activation

**DOI:** 10.3390/ijms21093309

**Published:** 2020-05-07

**Authors:** Hiroshi Matsuoka, Miyu Katayama, Ami Ohishi, Kaoruko Miya, Riki Tokunaga, Sou Kobayashi, Yuya Nishimoto, Kazutake Hirooka, Akiho Shima, Akihiro Michihara

**Affiliations:** 1 Laboratory of Genome Function and Pathophysiology, Faculty of Pharmacy and Pharmaceutical Sciences, Fukuyama University, Fukuyama, Hiroshima 729-0292, Japan; miyu.k0830@icloud.com (M.K.); cocoa-chocola.0217@docomo.ne.jp (A.O.); 51c.o_v.mii8k@ezweb.ne.jp (K.M.); ichiriki3672@outlook.jp (R.T.); sou0113com@gmail.com (S.K.); 0ms323593r9017u@au.com (Y.N.); a-shima@fukuyama-u.ac.jp (A.S.); mitihara@fukuyama-u.ac.jp (A.M.); 2 Department of Biotechnology, Faculty of Life Science and Biotechnology, Fukuyama University, Fukuyama, Hiroshima 729-0292, Japan; hirooka@fukuyama-u.ac.jp

**Keywords:** Alzheimer’s disease, cholesterol metabolism, oxysterols, 24S-hydroxycholesterol, CYP39A1, retinoic acid receptor-related orphan receptor α, intronic response element

## Abstract

Oxysterols, important regulators of cholesterol homeostasis in the brain, are affected by neurodegenerative diseases. Early-onset Alzheimer’s disease is associated with higher levels of circulating brain-derived 24S-hydroxycholesterol (24S-OHC). Conversion of cholesterol to 24S-OHC is mediated by cholesterol 24S-hydroxylase in the brain, which is the major pathway for oxysterol elimination, followed by oxidation through hepatic first-pass metabolism by CYP39A1. Abnormal CYP39A1 expression results in accumulation of 24S-OHC, influencing neurodegenerative disease-related deterioration; thus, it is important to understand the normal elimination of 24S-OHC and the system regulating CYP39A1, a selective hepatic metabolic enzyme of 24S-OHC. We examined the role of transcriptional regulation by retinoic acid receptor-related orphan receptor α (RORα), a nuclear receptor that responds to oxysterol ligands. In humans, the promoter and first intronic regions of CYP39A1 contain two putative RORα response elements (ROREs). RORα binding and responses of these ROREs were assessed using electrophoretic mobility shift, chromatin immunoprecipitation, and luciferase reporter assays. CYP39A1 was upregulated by RORα overexpression in HEK293 cells, while RORα knockdown by siRNA significantly downregulated CYP39A1 expression in human hepatoma cells. Additionally, CYP39A1 was induced by RORα agonist treatment, suggesting that CYP39A1 expression is activated by RORα nuclear receptors. This may provide a way to increase CYP39A1 activity using RORα agonists, and help halt 24S-OHC accumulation in neurodegenerative illnesses.

## 1. Introduction

While the brain only accounts for 2% of the total body weight, it contains 25% of all the cholesterol in the body [1]. The balanced removal of oxysterol through cholesterol oxidation regulates cholesterol homeostasis in the brain. Removal of cholesterol from brain tissue is difficult, but it can cross the blood-brain barrier (BBB) following oxidative conversion to oxysterol [2]. In the brain, cholesterol oxidation occurs by either reactive oxygen species [3] or enzymes, including CYP46A1, CYP27A1, and CH25 l [2]; about 70% of enzymatic oxidation is performed by CYP46A1. About 2–7 mg of cholesterol gets converted to 24S-hydroxycholesterol (24S-OHC), moving from the brain to the periphery every 24 h [4], where it is selectively transformed by the 7-α-hydroxylase activity of CYP39A1, a cytochrome P450 enzyme involved in hepatic first-pass metabolism [5].

27-OHC is the main oxysterol that flows from the periphery of the blood circulation into the brain through the BBB [6]. The 27-OHC accumulation is associated with hypercholesterolemia and oxidative stress, risk factors for cognitive loss, and 27-OHC is increased in the brains of patients with Alzheimer’s disease (AD) [7,8]. Some of the mechanisms by which 27-OHC may exert its harmful effect have been elucidated, at high concentrations. For example, AD-like pathology results from the induction of endoplasmic reticulum stress and activation of the C/EBP homologous protein [9], and the regulation of neuronal cell death dependent on phosphorylation of forkhead box protein O1, in a concentration-dependent manner [10]. In addition, 24S-OHC accumulation due to abnormal expression of CYP46A1 and CYP39A1 results in neuronal death, contributing to Alzheimer’s disease (AD)-related neurodegeneration [11,12]. Investigations into the molecular mechanisms regulating the association between cholesterol and AD identified an apolipoprotein E gene variant as a major genetic AD risk factor, consistent with a role for cholesterol in AD pathogenesis [13]. Over the course of AD-associated neurodegeneration, cell membranes degrade, releasing cholesterol into the extracellular space. The 24S-OHC concentrations in AD patients with dementia are modestly higher than in healthy controls [14]. Moreover, high concentrations of 24S-OHC induce a nonapoptotic programmed cell death in neurons [15]. Accumulation of amyloid-β peptide (Aβ) is believed to be the earliest observable event in AD development; 24S-OHC enhances Aβ accumulation by increasing amyloid precursor protein expression in neuroblastoma cells [16]. Plasma 24S-OHC concentrations depend upon opposing cerebral production–hepatic elimination processes and the number of metabolically active neurons in the brain, and 24S-OHC is an AD biomarker [17]. By regulating CYP39A1 activity, 24S-OHC accumulation may be inhibited, delaying AD-related neurodegeneration; however, the transcriptional regulation systems affecting CYP39A1 expression remain obscure.

Dual molecular effects of retinoic acid receptor-related orphan receptor α (RORα) dominant mutations cause two variants of syndromic intellectual disability, resulting in either autism or cerebellar ataxia [18]. Gene expression analyses of AD-affected brains and modeling of computationally-derived clusters and modules within networks identified strong ties between RORα and genes involved in AD etiology. Functional mapping schemes based on activity and interaction data affirmed this network links to RORα, highlighting its probable central role in driving AD pathology/etiology [19]. RORα nuclear receptors are essential for cerebellar development, and are involved in regulating several cellular processes, including circadian rhythm maintenance and lipid metabolism [20]. The spontaneous staggerer mutant mouse, a degenerative cerebellar model in which animals become ataxic due to neurodegeneration of Purkinje cells resulting from impaired dendritic development, was discovered to result from homozygous, intragenic RORα deletions [21]. RORα overexpression protects neurons against oxidative stress-induced apoptosis [22], and RORα regulates gene transcription by binding its respective response elements (RORE; a consensus AGGTCA motif preceded by an A/T-rich sequence) as monomers upon oxysterol ligand binding. 24S-OHC, as an oxysterol, is a CYP39A1 substrate that acts on RORα and RORγ [23]. Furthermore, 24S-OHC functions as an RORα/γ inverse agonist, suppressing the constitutive transcriptional activity of these receptors. Additionally, 24S-OHC suppress the expression of several RORα target genes in an ROR-dependent manner, and decreases the ability of RORα to recruit the coactivator SRC-2 when bound to the RORα target promoter [24]. RORα directly regulates cytochrome P450 enzymes, including CYP7B1 [25] and CYP8B1 [26]. Possible gene therapies for treating neurological diseases by administration of synthetic and endogenous ligands for RORα have been suggested, including CYP modulation [27].

CYP39A1 is expressed mainly in the liver, is subject to feedback regulation by sterols, and is thought to be regulated in conjunction with receptors and enzymes in cholesterol supply pathways, including the low-density lipoprotein receptor and 3-hydroxy-3-methylglutaryl-coenzyme A (HMG-CoA) reductase and synthase. CYP39A1 mRNA expression is upregulated upon dietary cholesterol intake in rats [28]. This response predicts the existence of regulatory elements in the promoter of the gene which respond in a positive fashion to cholesterol. Such elements may or may not differ from the sterol regulatory elements identified in genes coding for HMG-CoA synthase and others in the cholesterol synthesis pathways which respond negatively to cholesterol [29]. However, the transcriptional and inducible regulatory systems controlling the CYP39A1 gene are poorly understood. In this study, we aimed to elucidate the role of RORα and investigate the possibility of inducing CYP39A1 activity by RORα agonism.

## 2. Results

### 2.1. RORα Bound to ROREs of CYP39A1 Promoter and Intronic Regions

**Figure 1:**
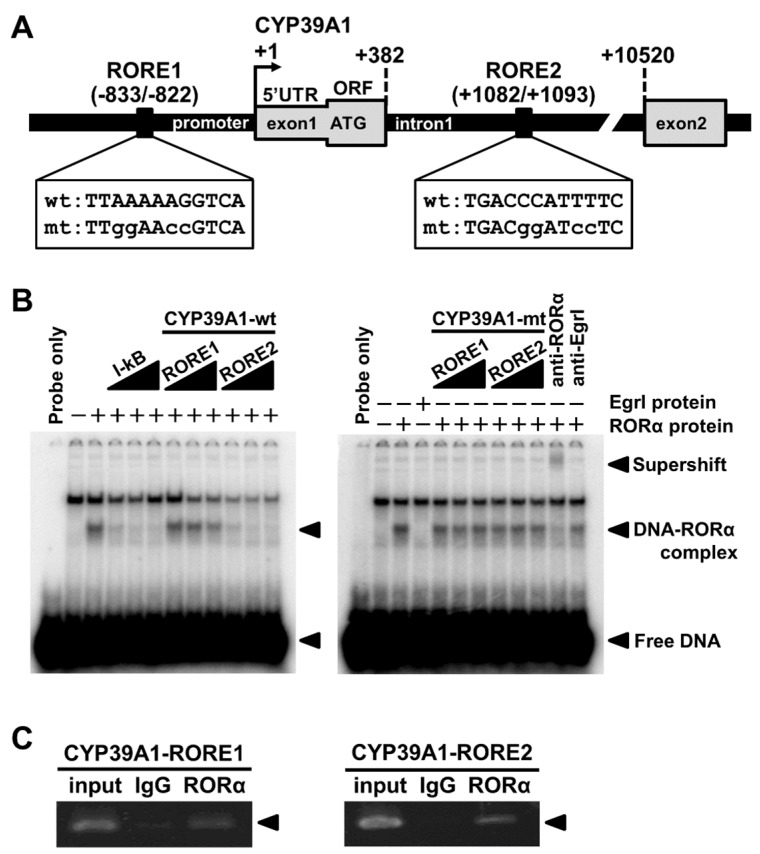
CYP39A1 activates retinoic acid receptor-related orphan receptor α (RORα) through direct binding of RORα response elements (ROREs) of the CYP39A1 promoter. (**A**) The promoter region of CYP39A1. Two predicted ROREs are indicated, along with the arrangement of RORE1 and RORE2 and their nucleotide sequences, which were used for mutation analyses. Mutated bases are indicated by lowercase characters. Upstream elements are indicated by a minus sign; downstream elements are indicated by a plus sign relative to the transcription start site (TSS), identified as +1. UTR; untranslated region, ORF; open reading frame. (**B**) Electrophoretic mobility shift assays (EMSAs) showing in vitro binding results of RORα interacting with ROREs of the CYP39A1 promoter. DNA oligonucleotides containing RORα binding sites were end-labeled with [γ-^32^P] competitor DNA oligonucleotides of IκB, CYP39A1-RORE1 or -RORE2, and incubated with RORα extracts translated in vitro. For EMSAs, anti-RORα antibodies were added to each reaction; a negative control of EgrI, a transcription factor that does not bind to ROREs, was added for comparison with anti-EgrI antibodies. (**C**) Chromatin immunoprecipitation (ChIP) assays with the anti-RORα antibody showing in vivo binding results of RORα with the ROREs. PCR was performed using primers for two RORE-containing regions in the CYP39A1 promoter. Normal rabbit IgG was used as a negative control.

Electrophoretic mobility shift assays (EMSAs) were used to assess RORα binding to DNA of two putative ROREs of the promoter and first intron of CYP39A1 (RORE1: −833/−822 as the upstream region; RORE2: +1082/+1093 as the downstream region; Figure 1A). In binding assays with RORE1 and RORE2 competing with IκB oligonucleotides, known ROREs that bind RORα, binding between IκB and RORα was inhibited. In contrast, RORE1 and RORE2 competition following a base substitution mutation did not inhibit binding of IκB DNA to RORα. Supershift experiments with the anti-RORα antibody showed specific binding of RORα to ROREs. Thus, RORα bound to CYP39A1 RORE1 and RORE2; of these, RORE2 and RORα had the highest binding affinity (Figure 1B). Complexes containing RORα and RORE in the promoter and first intronic regions of the CYP39A1 gene were assessed using ChIP-PCR in HepG2 cells to identify RORE–RORα complexes that could be introduced into cells. Following ChIP using an RORα antibody, we performed PCR so that RORE1 (−995/−724, upstream region) and RORE2 (+887/+1264, downstream intron region) sequences could be included, resulting in RORα binding for both RORE1 and RORE2 (Figure 1C).

### 2.2. RORα and RORE Responses in CYP39A1 Promoter and Intronic Regions

**Figure 2:**
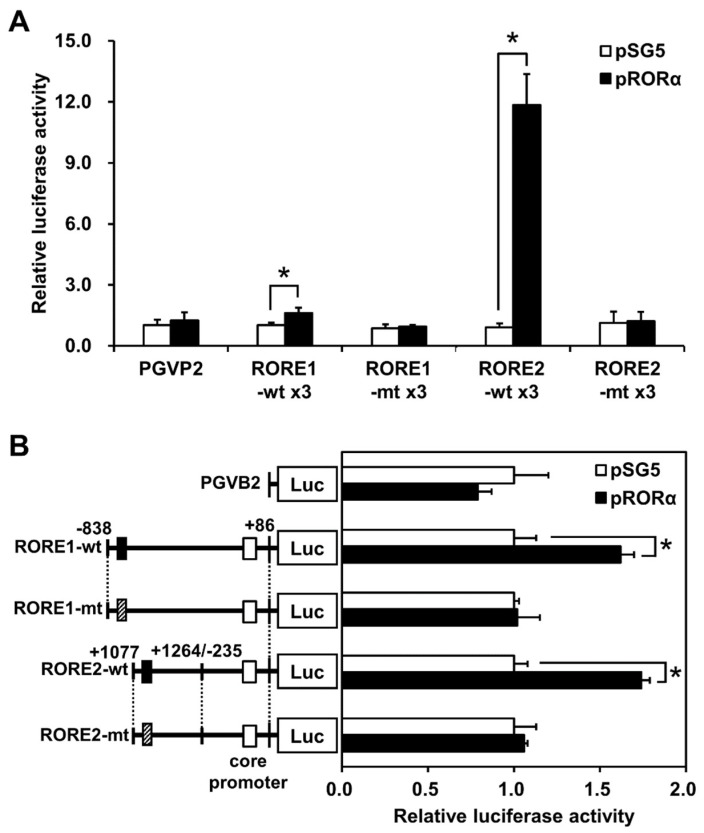
RORα induces CYP39A1 expression by directly interacting with ROREs of the CYP39A1 promoter. RORα expression and reporter assays were employed using constructs containing RORE1 and RORE2 sites in the promoter and intron regions of the CYP39A1 gene. The RORα expression vector (pRORα) and an empty vector (pSG5) were compared. (**A**) Luciferase assays showing the effect of RORα on luciferase reporter gene activities of constructs containing direct triplet repeats of wild-type (Wt) or mutant-type (Mt) ROREs of the CYP39A1 gene. Data are pooled from three independent experiments. * *p* < 0.05. (**B**) Luciferase assays showing effects of RORα on the reporter gene expression of constructs containing Wt or Mt RORE1 or RORE2 regions linked to the core promoter of the CYP39A1 gene. Data are presented as means ± standard error of the means (*n* = 3). * *p* < 0.05.

Luciferase reporter assays were performed to estimate responses to RORα at the CYP39A1 ROREs. The reporter vector connected direct repeats of RORE1 or RORE2 to the upstream elements of the minimal SV40 promoter sequence. Responses were indicated by upregulation of RORE1 (1.6-fold) and RORE2 (11.8-fold). The reporter response disappeared following a base substitution (Figure 2A). A vector that connected the CYP39A1 region and RORE1 (−1219/+86) or RORE2 (+887/+1264 linked to −235/+86 as the core promoter region) to the upstream luciferase reporter region was built, then cells were transfected with the RORα expression vector. When RORE1 or RORE2 was included, a RORα response was observed; this response disappeared when a base substitution was introduced into the reporter region (Figure 2B).

### 2.3. CYP39A1 Expression Increased in Cells Overexpressing RORα

**Figure 3:**
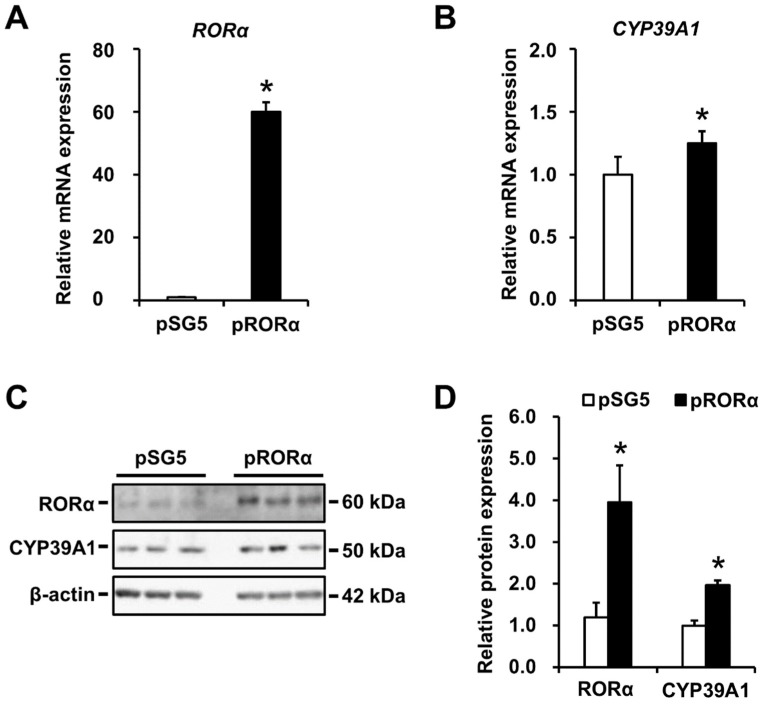
Regulation of endogenous CYP39A1 expression by RORα overexpression. RORα expression (pRORα) and empty vectors (pSG5) were transfected into HEK293 cells at 48 h; the expression of RORα (**A**) and CYP39A1 (**B**) mRNA transcripts were measured using qRT-PCR. (**C**) pRORα and pSG5 vectors were transfected into HEK293 cells at 72 h; the expression of RORα and CYP39A1 proteins were measured by western blot analysis. (**D**) Densitometric analysis of protein bands from RORα overexpression experiments quantified using a CS analyzer software. Data are presented as means ± standard error of the means (*n* = 3). * *p* < 0.05.

To quantify changes in expressions of CYP39A1 mRNA and proteins in cells overexpressing RORα, qRT-PCR and immunoblotting analyses were performed, respectively, in HEK293 cells. Changes in RORα proteins were measured. RORα and CYP39A1 mRNA levels increased in cells overexpressing RORα compared with the pSG5 empty vector (Figure 3A,B). Moreover, a 1.96-fold increase in CYP39A1 protein levels was observed, with a 3.95-fold increase in RORα protein levels (Figure 3C,D).

### 2.4. CYP39A1 Expression Decreased Following RORα Knockdown

**Figure 4:**
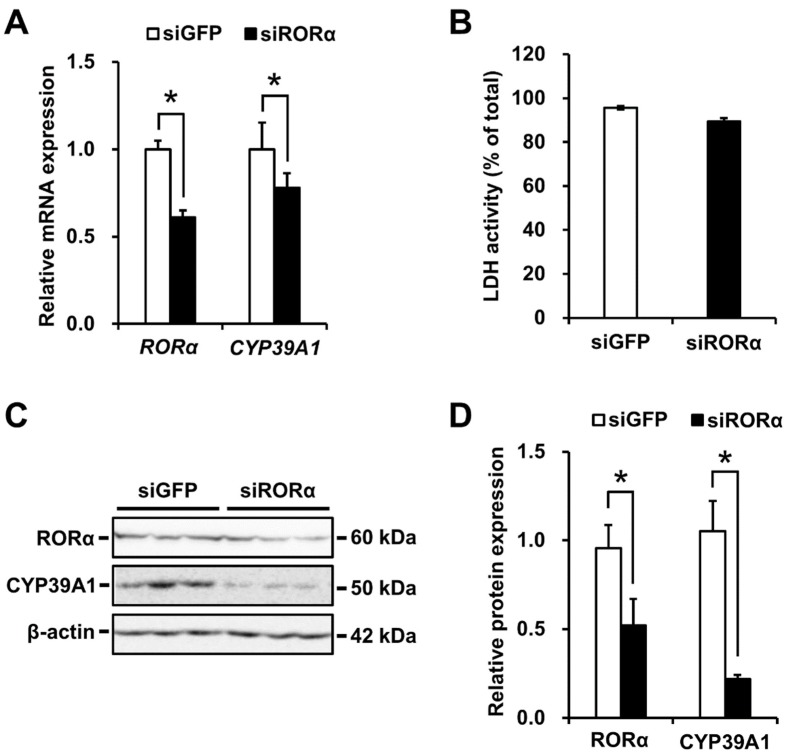
Regulation of endogenous CYP39A1 expression by RORα knockdown. (**A**) siRNAs of RORα gene (siRORα) and green fluorescent protein gene (siGFP) as a negative control were transfected into HepG2 cells at 48 h, then RORα and CYP39A1 mRNA expression levels were measured by qRT-PCR. (**B**) Effects of siRNA transfections on cell viability were estimated by measuring lactate dehydrogenase (LDH) activity (% of total including cells and medium) in the siRNA-treated cells. (**C**) siRORα and siGFP, for siRNA-induced knockdowns, were transfected into HepG2 cells at 48 h, then expression levels of RORα and CYP39A1 proteins were measured by western blot analysis. (**D**) Densitometric analysis of the protein bands from RORα knockdown quantified using a CS analyzer software. Data are presented as means ± standard error of the means (*n* = 3). * *p* < 0.05.

Silencing of the RORα gene by siRNA was performed to determine the impact of decreased RORα levels on CYP39A1 mRNA and protein concentrations in HepG2 cells. A decrease in CYP39A1 mRNA levels was observed following RORα knockdown (Figure 4A), and this expression was not decreased by siGFP as a negative control. Lactate dehydrogenase levels were measured as indicators of cell toxicity in the siRNA-transfected cells. The proportion of LDH in the intracellular compartment of siROR-treated cells was similar to that in the siGFP-treated cells. No cell toxicity resulted from siRNA knockdown (Figure 4B). A 0.5-fold decrease in RORα protein concentration resulted in decreased CYP39A1 protein concentration by 0.2-fold (Figure 4C,D).

### 2.5. CYP39A1 Expression Increased upon RORα Ligand Administration

**Figure 5:**
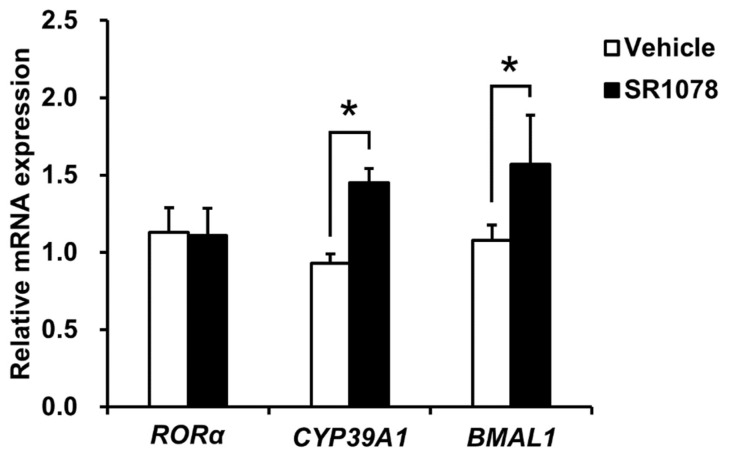
Effect of RORα agonist activation on CYP39A1 expression. HepG2 cells expressing endogenous RORα were treated without (vehicle) or with 5 μM SR1078, a synthetic RORα agonist, for 48 h; RORα, CYP39A1, and BMAL1 gene expression levels were quantified by qRT-PCR. Expression levels of each RORα target gene stimulated by the RORα agonist are presented as fold-changes relative to changes induced by vehicle alone. Data are presented as means ± standard error of the means (*n* = 3). * *p* < 0.05.

To investigate whether the synthetic RORα agonist, SR1078, would induce CYP39A1 mRNA expression, CYP39A1 mRNA levels in HepG2 cells treated with or without SR1078 were analyzed using qRT-PCR. Robust induction of CYP39A1 mRNA expression was observed in HepG2 cells following SR1078 administration (Figure 5). RORα expression was unchanged and BMAL1 expression, a positive control, was induced by SR1078.

## 3. Discussion

Oxysterol accumulation in the brain causes neuronal death in AD [11]. Almost all brain oxysterol is in the form of 24S-OHC, which can pass through the BBB for elimination through the circulatory system [4], involving metabolism by the hepatic CYP39A1 oxidizing pathway [5,30]. The 24S-OHC accumulation is prevented by the normal expression and function of CYP39A1; however, CYP39A1 regulation remains understudied, including its inducible nuclear receptor ligands. We showed, for the first time, that the nuclear receptor RORα regulates CYP39A1 expression levels in human hepatoma cells, which can be induced by SR1078, a RORα agonist. SR1078 also acts as an agonist of RORγ [31]. Therefore, CYP39A1 may be regulated by activation of RORα and RORγ.

RORα regulates transcription by binding its ROREs as monomers upon oxysterol ligand binding. In silico modeling revealed the promoter and first intronic regions of the human CYP39A1 gene contained two putative ROREs (Figure 1A). RORα bound and responded to the CYP39A1 ROREs, as determined by in vitro EMSAs (Figure 1B), and in cellulo ChIP (Figure 1C) and luciferase reporter assays (Figure 2). The first intronic region of the CYP39A1 gene contains RORE enhancers as transcription activation sites. Some introns contain enhancer elements or alternative promoters, while others elevate mRNA expression by an alternative intron-mediated enhancement process [32]. Here, RORE2 in the first intronic region, as well as RORE1 in the promoter region, upregulated CYP39A1 transcription. Moreover, CYP39A1 was upregulated through RORα overexpression in HEK293 cells (Figure 3), while RORα knockdown by siRNA significantly downregulated CYP39A1 expression in human hepatoma cells (Figure 4), suggesting that CYP39A1 expression is activated via RORα nuclear receptors. It has been reported that RORγ, involved in the transcriptional regulation of lipid metabolic genes, also acts as a transcription factor in the ROREs [33,34,35]. Therefore, RORγ may also act synergistically on the regulation of RORα. Whether CYP39A1 is involved in the regulatory mechanism of these two transcription factors remains to be elucidated.

The 24S-OHC molecule, a CYP39A1 substrate, acts on RORα and liver X receptors (LXRs) involved in oxysterol synthesis. Oxysterols, including 24S-OHC, 25-OHC, and 27-OHC are endogenous ligands for both RORα and LXRs [23]. RORα- and LXR-deficient mouse studies have revealed a potentially important functional crosstalk between RORα and LXRs [25]. LXRs possibly act as the major target of oxysterols, especially in the regulation of cholesterol metabolism, by binding to their respective response elements (LXREs, which contain two direct repeats of the consensus AGGTCA sequence, separated by four nucleotides) via the formation of heterodimers with an obligate partner, the retinoid X receptor [36]. Both nuclear receptors recognize response elements, including a commonly conserved half-site, but target genes of transcriptional regulation are not perfectly homologous [37]. Multiple RORα and LXR isoforms may exist or CYP39A1 may be regulated by response elements further upstream.

RORα and its inverse processes, including REV-ERB (reverse orientation the c-erbA-1 gene) nuclear receptors, may be targeted using synthetic ligands for treating several diseases [27], including atherosclerosis [38,39], nonalcoholic steatohepatitis (NASH) [40], and autism [41]. We showed that CYP39A1 was induced following RORα agonist treatment (Figure 5), suggesting CYP39A1 expression can be upregulated, increasing elimination and inhibiting accumulation of 24S-OHC, thus reducing neuronal death. Another possibility is that 24S-OHC acts as an endogenous inverse agonist at RORα and RORγ [24] and an agonist at LXR [18]. Others have reported that LXR does not act as an activator in the brain or liver in vivo, even if 24S-OHC production is increased in a transgenic mouse model with constitutive cholesterol 24-hydroxylase expression [42]. Therefore, 24S-OHC may concentration-dependently influence ROR- and LXR-mediated gene expression, although the inducible mechanisms through which it may act as an endogenous CYP39A1 substrate are unknown. Although the in vivo action of 24S-OHC is unclear, hepatic CYP39A1 expression is induced by steroid analogs via nuclear receptor activation and may have neuroprotective effects.

Treatment with the thiazolidinedione class of peroxisome proliferator-activated receptor gamma (PPARγ) agonists, widely prescribed for treating type II diabetes mellitus, have been shown to significantly improve memory and cognition in patients with AD [43]. The PPARγ transcriptional network is regulated by RORα in hepatic lipid homeostasis [44], but the relationship between RORα activity and AD remains unclear at the clinical, cellular, and molecular levels.

Ultimately, we showed the RORα nuclear receptor directly bound to two ROREs located upstream of the promoter and downstream of the first intronic region of the CYP39A1 transcription start site, resulting in CYP39A1 expression regulation. Moreover, RORα agonist administration induced CYP39A1 expression, suggesting RORα agonists could be used to suppress neuronal death.

## 4. Materials and Methods

### 4.1. Cell Culture

HepG2 and HEK293 cells obtained from the American Type Culture Collection (Manassas, VA, USA) were maintained in Dulbecco’s modified Eagle’s medium (DMEM) supplemented with 10% fetal bovine serum, 100 U/mL penicillin, and 100 µg/mL streptomycin at 37 °C and 5% CO_2_.

### 4.2. Electrophoretic Mobility Shift Assay (EMSA)

EMSAs were performed as described previously [45]. RORα was synthesized in vitro using the TNT Quick Coupled Transcription/Translation System (Promega, Madison, WI, USA) and incubated with reaction mixture on ice in the absence or presence of a double-stranded RORE oligonucleotide. After 20 min, 0.02 pmoles of a ^32^P-labeled double-stranded RORE probe of the NF-κB inhibitor (IκB) gene, a known RORα target [46], was added, and incubated at 30 °C. For competition assays, 5-, 10-, or 20-fold excess concentrations of unlabeled competitor oligonucleotides (two ROREs of CYP39A1-wild-type [wt] or mutant type [mt]) were added before the labeled oligonucleotides. For supershift assays, specific antibodies for anti-RORα (sc-28612; Santa Cruz Biotechnology, Santa Cruz, CA, USA) or anti-EgrI (as negative controls; sc-110; Santa Cruz Biotechnology) were added prior to the radiolabeled probe. Mixtures were subjected to 4% nondenaturing polyacrylamide gel electrophoresis in 0.5× TBE (tris/borate/ethylenediaminetetraacetic acid) buffer, visualized by autoradiographic imaging on a Typhoon 9400 (GE Healthcare, Little Chalfont, UK). Table S1 shows the probe sequences.

### 4.3. Chromatin Immunoprecipitation (ChIP)

Confluent HepG2 cells in 10 cm dishes were treated at room temperature with 37% formaldehyde and 2.5 M glycine for 10 min each. Cells were washed with 1× phosphate-buffered saline (PBS) (pH 7.4), scraped, and processed for chromatin preparation using OneDay ChIP Kits (Diagenode, Liege, Belgium), an anti-RORα antibody (sc-28612; Santa Cruz Biotechnology), and nonimmunized IgG as a negative control. RORE1- or RORE2-containing regions of CYP39A1 were PCR-amplified from immunoprecipitated DNA (primers shown in Table S1). Ethidium bromide-stained PCR products were run on 2% agarose gels, visualized using a CS Analyzer software (Atto, Tokyo, Japan).

### 4.4. Luciferase Reporter Assays

Sixteen hours before transfection, HepG2 cells were seeded in 24-well plates (0.5 × 10^5^ cells/well). Luciferase reporter plasmids, pRORE1-wt × 3 or pRORE2-wt × 3 carrying three RORE1 or RORE2 regions of CYP39A1 connected to the minimal SV40 promoter region in a PGV-P2 vector (Toyo Ink, Tokyo, Japan), or pCYP39A1-RORE1-wt (from −838 to +86 relative to the transcription start site [TSS]) or pCYP39A1-RORE2-wt (from +1077 to +1264 connected to the core promoter region, from −235 to +86 relative to the TSS) carrying the human CYP39A1 promoter and first intronic regions in a PGV-B2 vector (Toyo Ink) were cotransfected with expression vectors for RORα using an empty pSG5 vector (Agilent Technologies, Santa Clara, CA, USA), along with an internal β-galactosidase standard (each with 100 ng added/well), using Lipofectamine 2000 (Life Technologies, Gaithersburg, MD, USA) [45]. Reporter construct primers are shown in Table S1. Luciferase constructs, including RORE1 or RORE2, were mutated by a base substitution and compared to wt constructs. Cells were incubated for 32 h, then subjected to luciferase reporter assays, normalized relative to β-galactosidase activities, using PicaGene Luminescence Kits (Toyo Inc, Tokyo, Japan).

### 4.5. Quantitative Reverse Transcription-PCR (qRT-PCR)

Total RNA was extracted using ISOGEN kits (Nippon Gene, Tokyo, Japan). cDNA synthesis and qRT-PCR were performed as previously described [45]. Briefly, 300 ng of total RNA was reverse transcribed using random hexamers (Takara Bio) and Moloney murine leukemia virus reverse-transcriptase (Thermo Fisher Scientific). Of each cDNA, 10 ng was added to the SYBR Green Realtime PCR Master Mix (Toyobo, Osaka, Japan) or LightCycler 480 SYBR Green I Master (Roche, Basel, Switzerland) with 1 µM of each primer (see Table S1). Real-time fluorescence monitoring was performed using LightCycler 2.0 or LightCycler 480 II (Roche). Values were normalized to 18S rRNA, expressed relative to controls (treated empty vector (pSG5), small interfering RNA (siRNA) targeting green fluorescent protein (siGFP), or vehicle). The BMAL1 gene, a known RORα target, was used as a positive control.

### 4.6. Western Blotting

Cells were lysed in ice-cold lysis buffer (50 mM Tris-HCl, 1 mM EDTA, 200 mM sucrose, 1 µg/mL pepstatin, 1 µg/mL leupeptin, 0.5 mM phenylmethylsulfonyl fluoride, 1% SDS). Equal amounts of protein extracts were resolved by SDS-polyacrylamide gel electrophoresis and electrotransferred onto Immobilon-P membranes (Merck Millipore, Billerica, MA, USA). Detection and analyses using rabbit anti-RORα (sc-28612; Santa Cruz Biotechnology), anti-CYP39A1 (SAB4502119; Sigma-Aldrich, St. Louis, MO, USA), and anti-β-actin (60008; Proteintech, Chicago, IL, USA) were performed as previously described [47]. β-actin was used as endogenous internal controls to normalize gene expression. The band intensity was quantified using a CS analyzer software (Atto).

### 4.7. Overexpression Analysis

HEK293 cells (1 × 10^5^ cells/well) were seeded in 12-well plates 16 h before transfection. RORα expression plasmids with an empty pSG5 vector (0.3 µg/well for 12-well plates or 0.6 µg/well for 6-well plates) were transfected using Lipofectamine 2000 (Life Technologies); cells were harvested 48 or 72 h later. Total RNA and protein were extracted as described above.

### 4.8. siRNA Knockdown Analysis

RORα knockdown was performed with siRNA (siRORα), synthesized using an in vitro T7 transcription kit (Takara Bio) [34]. Similar methods were used to create negative control siGFP (Table S1 shows siRNA sequences). HepG2 cells were seeded in 24-well or 6-well plates (0.5 × 10^5^ or 2.0 × 10^5^ cells/well, respectively) 24 h before transfection. siRNAs were transfected using Lipofectamine 2000 (Life Technologies); cells were harvested 72 h later. Total RNA and protein were extracted as described above.

### 4.9. RORα Agonist Treatments

HepG2 cells were seeded (2.0 × 10^5^ cells/well) in 12-well plates in DMEM supplemented with 10% FBS, 100 µg/mL streptomycin, and 100 U/mL penicillin. After 24 h at 37 °C and 5% CO_2_, cells were treated without or with 5 µM SR1078 (RORα and RORγ agonist) [31] for 48 h. Total RNA was extracted as described above.

### 4.10. Statistical Analyses

Data are shown as means ± standard errors of the mean. Student’s t-tests were used to compare group means using Microsoft Excel software (version no. 1908; Microsoft corporation, Tokyo, Japan). *p* < 0.05 was considered statistically significant.

## Supplementary Materials

Supplementary materials can be found at https://www.mdpi.com/1422-0067/21/9/3309/s1. Table S1: Primers used in this study.

## Abbreviations

24S-OHC	24S-hydroxycholesterol
AD	Alzheimer’s disease
Aβ	amyloid-β peptide
BBB	blood brain barrier
ChIP	chromatin immunoprecipitation
DMEM	Dulbecco’s modified Eagle’s medium
EMSA	electrophoretic mobility shift assay
HMG-CoA	3-hydroxy-3-methylglutaryl-coenzyme A
IκB	inhibitor of nuclear factor-κB
LDH	lactate dehydrogenase
LXR	liver x receptor
mt	mutant-type
NASH	nonalcoholic steatohepatitis
REV-ERB	reverse orientation the c-erbA-1 gene
RORα	retinoic acid receptor-related orphan receptor α
RORE	RORα response element
PPARγ	peroxisome proliferator-activated receptor gamma
qRT-PCR	quantitative reverse transcription-PCR
siRNA	small interfering RNA
TSS	transcription start site
wt	wild-type
