# Supplementary material for: Orphan Nuclear Receptor RORα Regulates Enzymatic Metabolism of Cerebral 24S-Hydroxycholesterol through CYP39A1 Intronic Response Element Activation
Source: Int J Mol Sci. 2020 May 7;21(9):3309. doi: 10.3390/ijms21093309 (PMC7246805; doi:10.3390/ijms21093309)
Supplement: Supplementary file 1 [file ijms-21-03309-s001.pdf]

International Journal of Molecular Sciences

MDPI

Supplementary materials

Orphan nuclear receptor RORα regulates enzymatic metabolism of cerebral 24S-hydroxycholesterol through CYP39A1 intronic response element activation

Hiroshi Matsuoka, Miyu Katayama, Ami Ohishi, Kaoruko Miya, Riki Tokunaga, Sou Kobayashi, Yuya Nishimoto, Kazutake Hirooka, Akiho Shima and Akihiro Michihara

Table S1. Primers used in this study.

Primer	Sequence (5′–3′)
EMSA (Mutated sequences are underlined)	
CYP39A1-RORE1wt-sense	CGTTTTTAAAAAGGTCATCCTC
CYP39A1-RORE1wt-antisense	GAGGATGACCTTTTTAAAAACG
CYP39A1-RORE2wt-sense	TGTGGTGACCCATTTTCCATCT
CYP39A1-RORE2wt-antisense	AGATGGAAAATGGGTCACCACA
CYP39A1-RORE1mt-sense	CGTTTTTGGAACCGTCATCCTC
CYP39A1-RORE1mt-antisense	GAGGATGACGGTTCCAAAAACG
CYP39A1-RORE2mt-sense	TGTGGTGACGGATCCTCCATCT
CYP39A1-RORE2mt-antisense	AGATGGAGGATCCGTCACCACA
Ikb-RORE-sense	GATCCAATGTAGGTCACATG
Ikb-RORE-antisense	CATGTGACCTACATTGGATC
ChIP-PCR	
ChIP-CYP39A1-RORE1-FW	AGTAGTCGATGCTCTGTGAG
ChIP-CYP39A1-RORE1-RV	CTTCCCTTAGGCATGTGCTG
ChIP-CYP39A1-RORE2-FW	AGTAATCCTCCTTGTAGTAGATG
ChIP-CYP39A1-RORE2-RV	CAGTACAGCTGGTGTGATATTC
Luciferase reporter cloning (Mutated sequences are underlined)	
pCYP39A1-RORE1wt-MluI-FW	ACACGCGTCGTTTTTAAAAAGGTCATCCTC
pCYP39A1-RORE1mt-MluI-FW	ACACGCGTCGTTTTTGGAACCGTCATCCTC
pCYP39A1-RORE2wt-KpnI-FW	AATGGTACCTGTGGTGACCCATTTTCCATCTGG TTAGC
pCYP39A1-RORE2mt-KpnI-FW	AATGGTACCTGTGGTGACGGATCCTCCATCTGG TTAGC
pCYP39A1(−235)-MluI-FW	CTAACGCGTAGCTGGAGTGGTCCAGCTGCC
pCYP39A1(+1264)-MluI-RV	TAAAACGCGTGTACAGCTGGTGTGATATTC
pCYP39A1(+86)-XhoI-RV	GCAACTCGAGCAGCTCTCCTTCGTAACTGTAG
PGVB2-FW	GCCCAAGCTACCATGATAAG
PGVB2-RV	TCATAGCTTCTGCCAACCGAAC
qRT-PCR	
rtRORA-FW	TCCATGCAAGATCTGTGGAG
rtRORA-RV	ACAGCATCTCGAGACATCCC
rtCYP39A1-FW	TTGGACACTTGCATACGTCC
rtCYP39A1-RV	CTAGTAATGACACCAGGAGC
rtBMAL1-FW	GTTCTTCTATTCTTGGTGAGAAC

rtBMAL1-RV	ACAGCCATTGCTGCCTCATC
rt18S rRNA-FW	CGATAACGAACGAGACTCTGG
rt18S rRNA-RV	TAGGGTAGGCACACGCTGAGC
SiRNA experiments	(Synthesised siRNA sequences are underlined)
siRORA258-S-u	GATCACTAATACGACTCACTATAGGGGTCAGAAGAACTGTTTGATTT
siRORA258-S-d	AAATCAAACAGTTCTTCTGACCCCTATAGTGAGTCGTATTAGTGATC
siRORA258-AS-u	GATCACTAATACGACTCACTATAGGGATCAAACAGTTCTTCTGACTT
siRORA258-AS-d	AAGTCAGAAGAACTGTTTGATCCCTATAGTGAGTCGTATTAGTGATC
siRORA1388-S-u	GATCACTAATACGACTCACTATAGGGCTAATGGCATTTAAAGCAATT
siRORA1388-S-d	AATTGCTTTAAATGCCATTAGCCCTATAGTGAGTCGTATTAGTGATC
siRORA1388-AS-u	GATCACTAATACGACTCACTATAGGGTTGCTTTAAATGCCATTAGTT
siRORA1388-AS-d	AACTAATGGCATTTAAAGCAACCCTATAGTGAGTCGTATTAGTGATC
siGFP-S-u	GATCACTAATACGACTCACTATAGGGCAAGCTGACCCTGAAGTTCTT
siGFP-S-d	AAGAACTTCAGGGTCAGCTTGCCCTATAGTGAGTCGTATTAGTGATC
siGFP-AS-u	GATCACTAATACGACTCACTATAGGGGAACTTCAGGGTCAGCTTGTT
siGFP-AS-d	AACAAGCTGACCCTGAAGTTCCCCTATAGTGAGTCGTATTAGTGATC
